# Strain-enhanced giant Rashba spin splitting in ultrathin KTaO_3_ films for spin-polarized photocurrents

**DOI:** 10.1039/d0ra08745a

**Published:** 2020-12-15

**Authors:** Ning Wu, Xue-Jing Zhang, Bang-Gui Liu

**Affiliations:** Beijing National Laboratory for Condensed Matter Physics, Institute of Physics, Chinese Academy of Sciences Beijing 100190 China bgliu@iphy.ac.cn; School of Physical Sciences, University of Chinese Academy of Sciences Beijing 100190 China

## Abstract

Strong Rashba effects at semiconductor surfaces and interfaces have attracted great attention for basic scientific exploration and practical applications. Here, we show through first-principles investigation that applying biaxial stress can cause tunable and giant Rashba effects in ultrathin KTaO_3_ (KTO) (001) films with the most stable surfaces. When increasing the in-plane compressive strain to −5%, the Rashba spin splitting energy reaches *E*_R_ = 140 meV, corresponding to the Rashba coupling constant *α*_R_ = 1.3 eV Å. We investigate its strain-dependent crystal structures, energy bands, and related properties, and thereby elucidate the mechanism for the giant Rashba effects. Further calculations show that the giant Rashba spin splitting can remain or be enhanced when capping layer and/or Si substrate are added, and a SrTiO_3_ capping can make the Rashba spin splitting energy reach the record 190 meV. Furthermore, it is elucidated that strong circular photogalvanic effect can be achieved for spin-polarized photocurrents in the KTO thin films or related heterostructures, which is promising for future spintronic and optoelectronic applications.

## Abstract 2

Strong Rashba effects at semiconductor surfaces and interfaces have attracted attention for exploration and applications. We show with first-principles investigation that applying biaxial stress can cause tunable and giant Rashba effects in ultrathin KTaO_3_ (KTO) (001) films.

## Graphical abstract

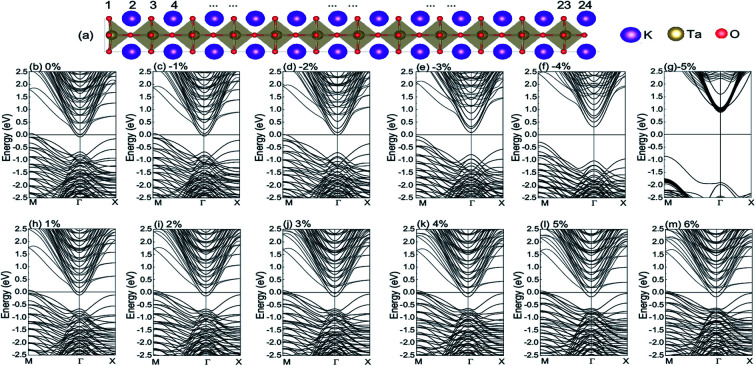

## I. Introduction

The Rashba spin–orbit interaction^1–3^ due to the broken inversion symmetry and the atomic spin–orbit coupling (SOC) can bring about the momentum-dependent spin splitting of the electron states. Rashba effect can play key roles in quantum wells,^4^ two dimensional (2D) electron gases (2DEG),^5^ and thin films based on traditional III–V semiconductors.^6^ After intensive investigations, one can tailor the Rashba coupling by electric field and strain, and design artificial microstructures for various applications. External electric field can be used to modulate the magnitude of Rashba spin splitting in LaAlO_3_/SrTiO_3_ (LAO/STO) interface^7^ and InSe multilayer.^8^ The Rashba spin splitting can be effectively tuned by varying the interlayer distance in graphene/As–I van der Waals heterostructure^9^ and adjusting the halogen doping concentration in doped PtSe_2_ monolayer.^10^ In addition, it is very interesting to manipulate the Rashba spin–orbit coupling by applying stress, as were done in 2D LaOBiS_2_,^11^ binary alloyed hexagonal nanosheets,^12^ 2D heterostructures,^13^ BiSb monolayer,^14^ and BiTeI monolayer.^15^ Large Rashba interaction can be used to realize spin polarization and spin injection by applying electric field^16,17^ and produce spin-polarized photocurrents through polarized light.^18–23^

Recently, a 2DEG was observed at KTaO_3_ (KTO) (100) surface^24,25^ in terms of the angle-resolved photoemission (ARPES) spectrum.^24^ For another 2DEG at an amorphous-LAO/KTO interface, an experimental analysis of the weak anti-localization effect resulted in a Rashba coupling constant 0.1 eV Å and a 50-fold enhanced Hall mobility of charge carriers.^26^ Surprisingly, hysteretic magnetoresistance up to 25 K and anomalous Hall effect up to 70 K were observed at an EuO/KTO interface.^27^ It was shown that LaVO_3_/KTO heterostructure with strong spin–orbits interactions can be used to produce planar Hall effect, anisotropic magnetoresistance, and 2DEG.^28,29^ Theoretically, the magnitude of Rashba spin splitting in a KTO surface was studied by applying external electric fields in a symmetric slab model.^30^ It is highly desirable to investigate the effects of in-plane strain fields on the strength of Rashba spin splitting for KTO surfaces. Actually, strain (stress) is a wonderful approach to manipulate the crystal structures of KTO and thus control their electronic structures and functional properties. Recent studies have demonstrated that the strain can affect the formation and migration of oxygen vacancies in KTO^31^ and induce electron–hole interchanging of the two opposite surface 2D carrier gases in KTO ultrathin film.^32^ It has been also shown, experimentally and theoretically, that the KTO (001) 1 × 1 surfaces terminated with KO and TaO_2_ are most stable compared to others.^33,34^ Therefore, structurally stable ultrathin KTO (001) films with the two stable surfaces can be manipulated to realize giant Rashba spin effects.

Here, through first-principles calculations and theoretical analyses, we investigate the in-plane strain dependencies of the structural features, intrinsic electrostatic potentials, band edges, carrier concentrations, carrier effective masses, and Rashba parameters of the ultrathin KTO (001) films. We show that their Rashba spin splitting can be controlled by applying biaxial stress, and giant Rashba spin splitting can be obtained by applying compressive biaxial stress, being able to reach 140 meV. In addition, we explore effects of capping layers and/or Si substrate on the giant Rashba spin splitting, and show that a STO capping can make the Rashba spin splitting reach the record 190 meV, as shown in Table 2. Such giant Rashba effects in the ultrathin KTO films can be used to generate spin-polarized photocurrents through circular photogalvanic effect.^18–23^ More detailed results will be presented in the following.

## II. Computational method and parameters

Our first-principles calculation is performed using the projector-augmented wave method within the density-functional theory,^35,36^ as implemented in the Vienna *Ab initio* Simulation Package (VASP).^37,38^ To describe the exchange–correlation energy, we used the general gradient approximation (GGA) with the Perdew–Burke–Ernzerhof for solids (PBEsol) parametrization.^39,40^ The on-site Coulomb interaction in 5d states of transition-metal ions is corrected by the DFT+*U* (where *U* is the Hubbard energy) method.^41^ The effective value *U*_eff_ = 3 eV is employed for Ta 5d states in this work, as it is well established that such a value is appropriate to describe these strongly-correlated states.^32^ An Monkhorst–Pack *k*-point grid of 4 × 4 × 1 is used for reciprocal space integration, and the plane wave energy cutoff is set to 500 eV. Our convergence standard requires that the Hellmann–Feynman force on each atom is less than 0.01 eV Å^−1^ and the absolute total energy difference between two successive consistent loops is smaller than 1 × 10^−5^ eV. A fully converged electronic structure is used for further calculation including SOC. Band calculations with SOC are confirmed with larger *k*-point grid 6 × 6 × 1 in cases of zero and −5% strain values, and the resulting changes are very small. A 20 Å thick vacuum layer is used in the KTO-slab geometry. Additional calculations with vacuum layer of 30 Å and dipole corrections^42^ are made for confirmation. When a biaxial stress is applied, the in-plane strain is defined as *ε*_s_ = (*a* − *a*_0_)/*a*_0_ × 100%, where *a*_0_ is the experimental lattice constant of bulk KTO without strain (*a*_0_ = 3.989 Å (ref. 43)) and *a* is the in-plane lattice constant of strained KTO slab. We also optimize cubic KTO bulk by using the above parameters and better convergence standards: *k*-point grid 15 × 15 × 15, the Hellmann–Feynman force 0.001 eV Å^−1^, and total energy 1 × 10^−8^ eV. This optimization produces the theoretical lattice constant of KTO: *a*_T_ = 3.996 Å. Because *a*_T_ is only 0.2% larger than *a*_0_, we use the experimental *a*_0_ as our reference when defining strain values. Given an in-plane strain value, the out-of-plane lattice and all the internal atomic positions are allowed to relax sufficiently during optimization.

## III. Results and discussion

### A. KTO slab under biaxial stress

**Fig. 1:**
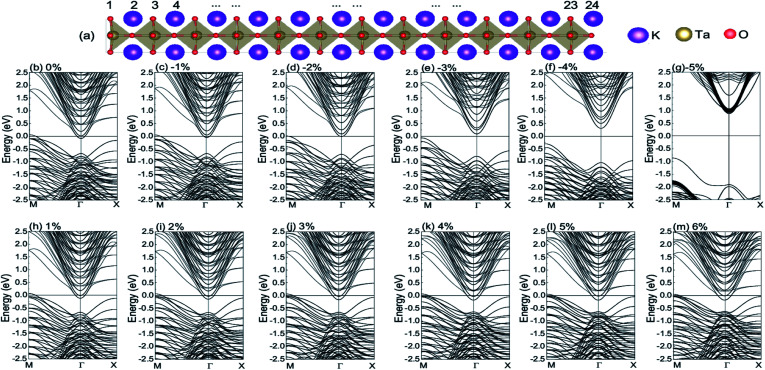
(a) Side view of the optimized atomic structure of the KTO ultrathin film (slab) at *ε*_s_ = 0%. (b–m) Band structures of the KTO ultrathin film at different strain values: ε_s_ = 0%, −1%, −2%, −3%, −4%, −5%, 1%, 2%, 3%, 4%, 5%, and 6%. The K, Ta, and O atoms are shown by the purple, yellow, and red balls, respectively. The Fermi level *E*_f_ is at the zero energy.

We construct a KTO slab model to describe the KTO ultrathin film under different biaxial stresses. The slab consists of *m* = 12 KTO unit cells along the vertical [001] axis. Fig. 1(a) shows the optimized structure of the KTO slab at the in-plane strain *ε*_s_ = 0% (zero stress). We study the strained KTO slabs with the in-plane strain *ε*_s_ ranging from −5% (compressive) to +8% (tensile). With a given in-plane strain, the system is fully optimized, with the out-of-plane strain being determined by requiring that the out-of-plane stress is zero, and thus we can determine the in-plane stress. Actually, this is a system with biaxial stress. With the condition that the out-of-plane stress is zero, however, the in-plane stress is determined by the in-plane strain. Therefore, for convenience, we shall use the in-plane strain *ε*_s_ to characterize the strained slabs in the following. It is confirmed that the dipole correction has little effect in these results. In Fig. 1(b–m), we plot the representative electronic band structures along *M* (π,π) → *Γ* (0,0) → *X* (0,π) of the optimized KTO slabs for *ε*_s_ = 0%, −1%, −2%, −3%, −4%, −5%, 1%, 2%, 3%, 4%, 5%, and 6%, respectively. Here, SOC is taken into account, and the *k* vector is in units of 1/*a*, where *a* is the calculated lattice constant of the strained KTO slab. When *ε*_s_ is larger than −2%, the KTO slab remains metallic. For the metallic state, there are electron carriers near the *Γ* point and hole carriers near the *M* point, and they form a 2DEG at the TaO_2_-terminated surface and a 2D hole gas (2DHG) at the KO-terminated surface. It should be pointed out that the electron concentration in the 2DEG is equivalent to the hole concentration in the 2DHG.^32^ There is a critical strain *ε*_s_ = −2%, as shown in Fig. 1(d). From Fig. 1(g, f and e), it is clear that the KTO slab is insulating for *ε*_s_ = −5%, −4%, and −3%. Therefore, there is a strain-driven metal–insulator transition (MIT) at *ε*_s_ = −2%. For the insulating phase, there is an indirect semiconductor gap increasing with |*ε*_s_ + 0.02|, with the conduction band minimum (CBM) being at *Γ* point and the valence band maximum (VBM) at the *M* point.

**Fig. 2:**
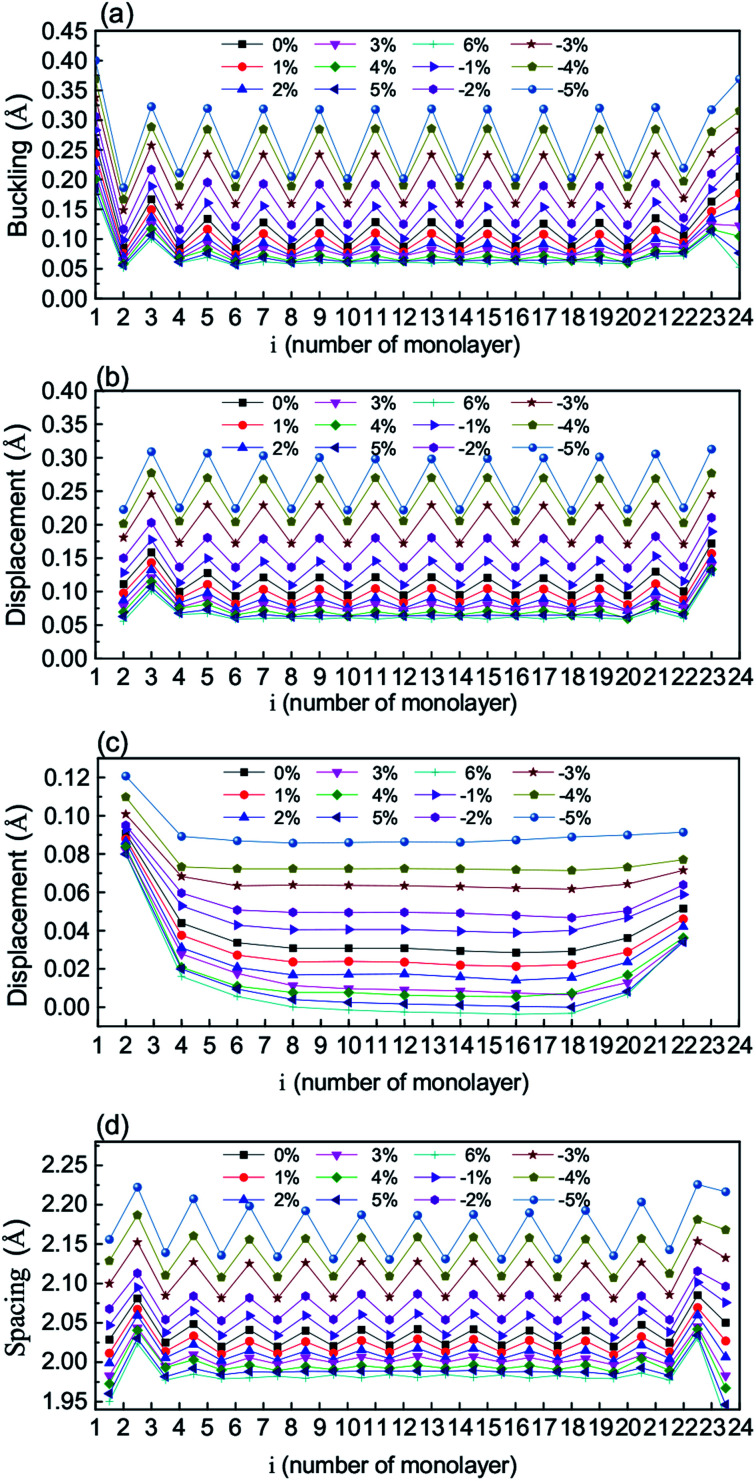
The monolayer-resolved intra-monolayer bucklings (a), displacements of cations (K, Ta) with respect to the nearest O anions (b), displacements of Ta with respect to the nearest K atoms (c), and inter-monolayer spacings (d) of the KTO slab at the different *ε*_s_ values.

To show the stress-driven structural features, we present in Fig. 2 the monolayer-resolved intra-monolayer ionic bucklings (*B*_i_, defined as the maximal cation–anion out-of-plane difference within the monolayer), out-of-plane cation displacements with respect to the centers of the nearest O anions (*D*^K^_i_ and *D*^T^_i_), out-of-plane Ta displacements with respect to the centers of the nearest K atoms (*D*^E^_i_, defined only for even i), and inter-monolayer spacings (*S*_i_) of the slab under the in-plane strain values between −5% and 6%. It is clear that the atomic positions of the surfaces change substantially with respect to the internal region in the four aspects, and in the internal region the four aspects are made nearly independent of monolayers at strong tensile strains. It is interesting that all the four values monotonically decrease with tensile strain, but increase with compressive strain. When the compressive in-plane strain becomes strong, the bucklings of the surface monolayers are substantially enhanced, and the displacements in the KO monolayers (the TaO_2_ monolayers) converge to a nearly constant value *d*_1_ (*d*_2_), with *d*_2_ > *d*_1_. By combining the buckling and spacing values, it is visible that the two surface single-unit-cells are a little separated from the main body, which is useful to reduce the effect of polarity and stabilize the slab structure. *D*^E^_i_ is frequently used in experimental measurement reflecting polarization. It is enhanced by compressive stress and reduced by tensile stress.

**Fig. 3:**
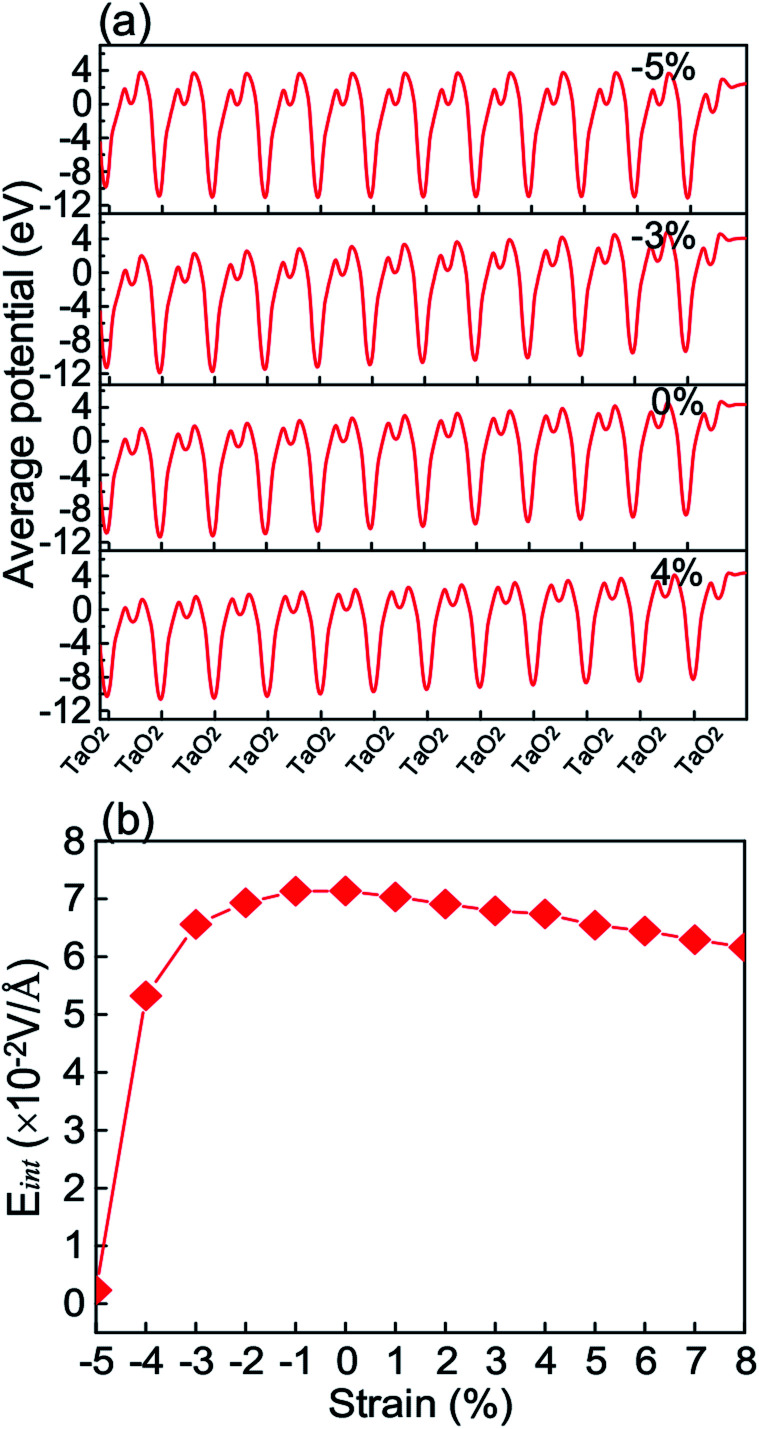
(a) The monolayer-resolved plane-averaged electrostatic potentials of the KTO slab for strain *ε*_s_ = −5%, −3%, 0%, and 4%, respectively. (b) The internal electric field (*E*_int_) as a function of the strain *ε*_s_.

Furthermore, we plot in Fig. 3(a) the plane-averaged electrostatic potentials for *ε*_s_ = −5%, −3%, 0, and 4% as representative strain values. It is clear that the maximal (or minimal) value increases from the left to right hand side in the cases of *ε*_s_ = −3%, 0, and 4%, but remains the same for *ε*_s_ = −5%. The internal electric field *E*_int_ can be estimated from the slope of the plane-averaged electrostatic potential shown in Fig. 3(a).^44^ The calculated results as a function of *ε*_s_ are presented in Fig. 3(b). The internal electric field at the unstrained KTO slab is 7.1 × 10^−2^ V Å^−1^, comparable with a previous study.^45^ It is clear that *E*_int_ slowly decreases with tensile strain, and accelerates with compressive strain, nearly reaching zero at *ε*_s_ = −5%. It is expected that the out-of-plane cation displacements with respect to the neighboring O atoms counteract the out-of-plane polarity of the KTO slab, which originates from the oppositely charged (TaO_2_)+ and (KO)− monolayers. When the compressive strain reaches *ε*_s_ = −5%, corresponding to the in-plane lattice constant 3.789 Å, the potential slope is almost diminished by the increasing polarization due to displacements. The large changes in *E*_int_ caused by strong compressive strains will change the energy bands.

### B. Energy band parameters

**Fig. 4:**
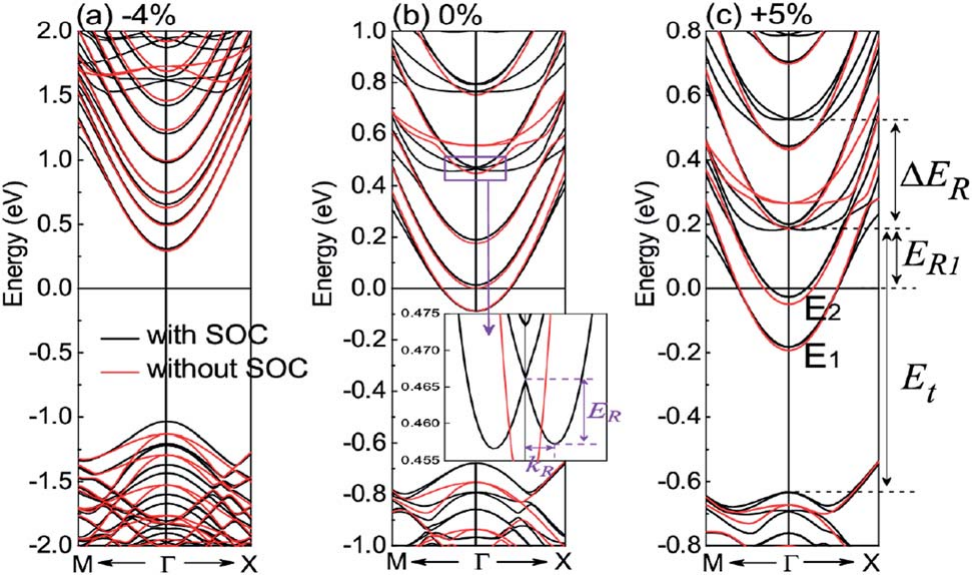
The band structures around *Γ* point of the KTO slab with (black) and without (red) SOC for *ε*_s_ = −4%, 0%, and 5% are magnified in (a), (b) and (c), respectively. The inset of (b) shows the definition of *E*_R_ and *k*_R_ used for estimating the Rashba parameters. The lowest and second lowest *d*_*xy*_ bands are labeled by *E*_1_ and *E*_2_.

To elucidate the band edges and electron concentrations for the strained KTO slab, we present in Fig. 4 the magnified electron band structures of the KTO slab near the *Γ* point for *ε*_s_ = −4%, 0%, and 5%. In the absence of SOC, the quantum confinement reduces the initial cubic symmetry of the Ta t_2g_ orbitals in the bulk perovskite. The triple degeneracy (excluding spin) of the t_2g_ bands at the *Γ* point is lifted, splitting the *d*_*xy*_ from *d*_*xz*_/*d*_*yz*_. When the inversion symmetry breaking is taken into account, the Ta atomic SOC further splits the *d*_*xz*_/*d*_*yz*_ bands into the upper part and the lower one. For the *ε*_s_ = 0% case shown in Fig. 4(b), only the lowest *d*_*xy*_ band is partially occupied and the band minimum (at the zone center) lies 0.089 eV below *E*_f_. The corresponding electron concentration of the 2DEG is 2.67 × 10^13^ cm^−2^, which is an order of magnitude smaller than 2 × 10^14^ cm^−2^ of the 2DEG formed at an experimental KTO surface from the ARPES measurements.^24^ This difference can be interpreted by the low formation energy for the oxygen vacancies at the KTO surface,^24,46^ which allows much more electrons in the 2DEG. For the *ε*_s_ = 5% case shown in Fig. 4(c), the minima of the occupied lowest and second lowest *d*_*xy*_ states (at the *Γ* point) are 0.182 and 0.027 eV below *E*_f_, in which the summed electron concentration is 6.98 × 10^13^ cm^−2^, larger than that in the unstrained system. For the *ε*_s_ = −4% case shown in Fig. 4(a), the lowest *d*_*xy*_ band lies 0.31 eV above the *E*_f_, which means that there are no carriers (without doping), in contrast with those of the *ε*_s_ = 0% and 5% cases.

It is obvious that there are some Rashba-like spin splitting in the conduction bands of the KTO slab for the *ε*_s_ between −5% and 8%. To further investigate the Rashba effects, we also present in Fig. 4 the magnified electron band structures with SOC of the KTO slab near the *Γ* point for *ε*_s_ = 0%.

**Fig. 5:**
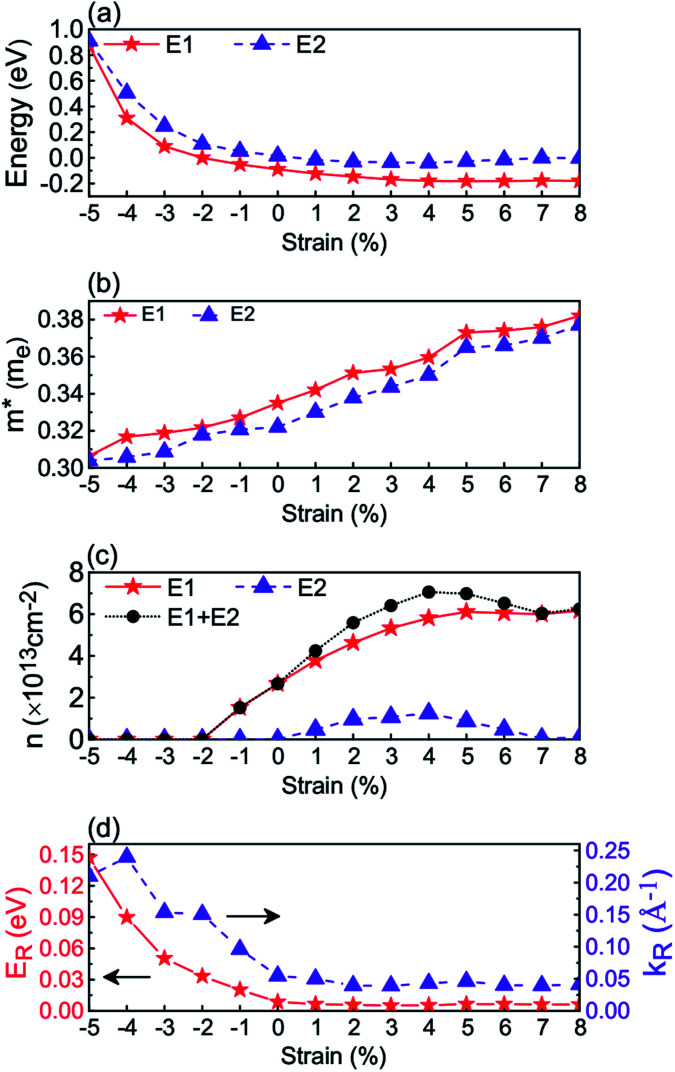
The strain *ε*_s_ dependencies of the energy positions of the *E*_1_ and *E*_2_ bands (a), the corresponding electron effective masses m* (b), the 2DEG concentration *n* (c), and the Rashba spin splitting energy *E*_R_ and *k*-vector offset *k*_R_ (d). The *E*_1_ and *E*_2_ bands are defined in Fig. 4.

To better describe the properties of the lowest and second lowest *d*_*xy*_ conduction bands near the *Γ* point respectively, defined by *E*_1_ and *E*_2_ in Fig. 4(c), we investigate and show the band edge positions, electron effective masses, and 2DEG concentrations of the *E*_1_ and *E*_2_ bands as functions of *ε*_s_ in Fig. 5. In Fig. 5(a), as *ε*_s_ changes from −5% to 4%, the band edge positions of *E*_1_ and *E*_2_ decrease rapidly with large compressive strain, but they change slowly with tensile strain. As *ε*_s_ varying from 5% to 8%, the band edge positions of *E*_1_ and *E*_2_ are almost unchanged. It should be noted that the bottom of the conduction band is less affected by the tensile strain, while it is significantly changed by the compressive strain. In detail, the band edge of the *E*_1_ band is below *E*_f_ for *ε*_s_ ≥ −1%, and that of *E*_2_ becomes below *E*_f_ for *ε*_s_ ≥ 1%.

In Fig. 5(b), the effective mass (m*) is evaluated from a second-order fit of the band energies using 
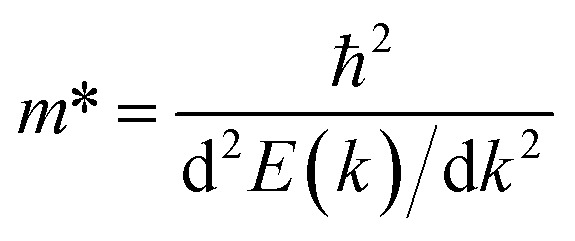
.^47^ Remarkably, the values of 
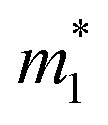
 for *E*_1_ and 
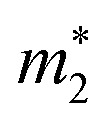
 for *E*_2_ in the unstrained KTO system are 0.35 and 0.32*m*_e_ (*m*_e_ is the mass of the free electron), respectively, which are both in excellent agreement with 0.30*m*_e_ for the KTO surface 2DEG measured by ARPES.^24^ When the strain changing from *ε*_s_ = −5% to 8%, 
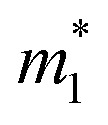
 and 
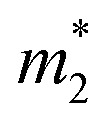
 increase, with 
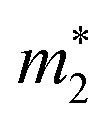
 being always smaller than 
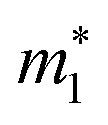
. Under *ε*_s_ = 8%, 
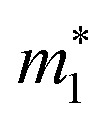
 and 
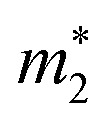
 reach the maximum values 0.38 and 0.37*m*_e_, respectively, which are still smaller than ∼0.5 and 0.6*m*_e_ recently determined for a surface 2DEG on STO.^48^ This suggests that developing high-mobility oxide electronics by KTO is better than by STO.

Fig. 5(c) shows the relationship between *ε*_s_ and the carrier concentrations. For *ε*_s_ ≤ −2%, the *E*_1_ and *E*_2_ bands are empty and the KTO film is insulating, which is consistent with the critical strain of insulator–metal transition shown in Fig. 1. The carrier concentrations *n*_1_ and *n*_2_ for the *E*_1_ and *E*_2_ bands have the maximum values at the *ε*_s_ = 5% and 4%, respectively, and the total 2DEG concentration *n* of the *E*_1_ + *E*_2_ bands reaches the maximum values of 7.06 × 10^13^ cm^−2^ at *ε*_s_ = 4%. This indicates that the conductivity of the 2DEG formed at the surface can be effectively modulated by the in-plane strain.

In addition, we summarize in Table 1 the energy differences (*E*_R1_ and *E*_t_) and the spin–orbit splitting energy Δ*E*_R_ between the lowest and the second lowest Rashba doublets, as defined in Fig. 4.

**Table 1:** The three band parameters [*E*_R1_, *E*_t_, and Δ*E*_R_ (eV)] and the two Rashba parameters [*E*_R_ (meV) and *k*_R_ (Å^−1^)] of the KTO ultrathin film at different strains

Strain	*E*_R1_	*E*_t_	Δ*E*_R_	*E*_R_	*k*_R_
*ε*_s_ = 5%	0.188	0.822	0.339	7	0.046
*ε*_s_ = 0%	0.466	1.144	0.298	9	0.054
*ε*_s_ = −1%	0.603	1.282	0.309	20	0.096
*ε*_s_ = −2%	0.810	1.516	0.309	33	0.151
*ε*_s_ = −3%	1.161	1.947	0.314	51	0.154
*ε*_s_ = −4%	1.618	2.652	0.325	90	0.24
*ε*_s_ = −5%	2.228	4.136	0.093	140	0.21

### C. Rashba spin splitting

Since the KTO slab obeys the *C*_4v_ point group symmetry, the symmetry-allowed linear spin-momentum coupling can be expressed as^3^*H*_R_ = *α*_R_(*k*_*x*_*σ*_*y*_ − *k*_*y*_*σ*_*x*_). According to the linear Rashba model, the dispersion due to the Rashba spin splitting can be described by1
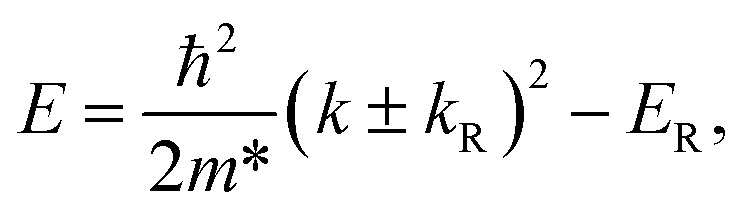
where *k* is the magnitude of the electron wave vector, m* is the electron effective mass, *E*_R_ = ℏ^2^*k*_R_^2^/2m* is the Rashba spin splitting energy, and *k*_R_ is the momentum offset *k*_R_ = 2*E*_R_/*α*_R_. The in-plane spin polarizations of the “+” and “−” eigenstates are oppositely aligned and normal to the electron wave vector. In the isotropic case, the Rashba coupling constant can be estimated by *α*_R_ = 2*E*_R_/*k*_R_, and *α*_R_ depends on the strength of SOC and inversion asymmetry.^49^

For the KTO slab, the lowest Rashba spin split bands near the *Γ* point are similar to those defined by eqn (1), and we present *E*_R_ and *k*_R_ in Fig. 5(d) for different in-plane strains. The calculated values of *E*_R_ and *k*_R_ are summarized in Table 1 for *ε*_s_ = 5%, 0%, −1%, −2%, −3%, −4%, and −5%. In Fig. 5(d), *E*_R_ and *k*_R_ increase drastically with the compressive strain increasing, but they both remain almost unchanged for increasing tensile strain. In Table 1, noticeably, *E*_R_ and *k*_R_ are 140 (90) meV and 0.21 (0.24) Å^−1^ for the KTO slab at *ε*_s_ = −5% (−4%). It is clear that compressive in-plane strain can enhance the Rashba spin splitting energy *E*_R_. Because *E*_int_ is near zero at *ε*_s_ = −5.0%, *E*_int_ becomes negative when *ε*_s_ < −5.0%, and consequently the conduction bands are reconstructed, which leads to smaller *E*_R_ or substantial deformation of the Rashba bands. Actually, this means that the maximal *E*_R_ is reached at *ε*_s_ = −5%.

To elucidate the mechanism responsible for the giant Rashba spin splitting, we investigate the strain-dependent structural parameters and electrostatic potentials. For the unstrained KTO slab, there is a strong intrinsic electric field *e*_0_ due to the out-of-plane alternate alignment of negative KO and positive TaO_2_ monolayers. When compressive biaxial stress is applied, there are out-of-plane displacements of cations with respect to the neighboring anions driven by the compressive in-plane strain *ε*_s_ and tensile out-of-plane strain, and in addition the ionic displacements cause an out-of-plane electric field *e*_d_ antiparallel to *e*_0_. Our calculated results show that *e*_d_ increases with *ε*_s_, reaching the maximum nearly at *ε*_s_ = −5%. Consequently, we can attribute the enhanced giant Rashba spin splitting energy to the strong intrinsic out-of-plane electric field *e*_d_ due to the large compressive biaxial stress.

For comparison, we summarize the *E*_R_, *k*_R_, and *α*_R_ values of some typical Rashba systems in Table 2. For brevity, we can take *E*_R_ as the key parameter to characterize such Rashba systems. *E*_R_ can reach 100 meV for BiTeI van der Waals bulk,^50^ or 190 meV for α-GeTe(111) film.^51^ In contrast, for perovskite oxides, the previous maximal *E*_R_ is 15 meV for KTaO_3_/BaHfO_3_ interface.^52^ It is clear that our strategy is very efficient to promote the Rashba spin splitting energy in perovskite oxides because our maximal *E*_R_ value reaches 140 meV at *ε*_s_ = −5%. Such large Rashba effect is useful to realize strong circular photogalvanic effect.^18–23^

**Table 2:** Rashba splitting energy *E*_R_ (meV), *k*-vector offset *k*_R_ (Å^−1^), and Rashba coupling constant *α*_R_ (eV Å) of typical 2D materials (monolayer and van der Waals multilayers), sp semiconductors, and perovskite oxides

System	*E*_R_	*k*_R_	*α*_R_
GaSe/MoSe_2_ van der Waals HS^13^	31	0.13	0.49
BiTeI monolayer (*ε*_s_ = 6%)^53^	55.7	0.054	2.05
BiTeI van der Waals bulk^50^	100	0.052	3.8
InAlAs/InGaAs interface^6^	<1.0	0.028	0.07
GeTe(111)/InP(111) interface^54^	5.403	0.010	1.08
α-GeTe(111) film^51^	190.0	0.13	4.2
BiAlO_3_ bulk crystal^55^	7.34	0.038	0.39
LaAlO_3_/SrTiO_3_ interface^7^	<5.0		0.01–0.05
KTaO_3_/BaHfO_3_ interface^52^	15		0.3
KTaO_3_ film (*ε*_s_ = −5%)	140	0.21	1.3
SrTiO_3_/KTaO_3_ bilayer (*ε*_s_ = −5%)	190	0.24	1.58

### D. Heterostructures and photogalvanic effect

Recent STM experiment and DFT calculation have already shown that the KTO (001) 1 × 1 surfaces terminated with KO and TaO_2_ are most stable compared to others.^33,34^ For more applications, the KTO films could be capped with STO overlayers, and/or should be grown on good substrates. The metallic interfaces can be obtained by generating carriers through experimentally applying gate voltage.^56^ Furthermore, the STO capping can change the Rashba spin splitting in the bare TaO_2_-terminated surfaces of KTO films. For the in-plane strains of −3%, −4%, and −5%, our calculations show that the maximal Rashba spin splitting energies are 25, 95, and 190 meV, respectively, and the corresponding *k* vector offsets are 0.12, 0.19, and 0.24 Å^−1^. It is a surprise that the STO capping can make the Rashba spin splitting enhanced to 190 meV at strain of −5%! We also study STO/KTO/Si trilayer to simulate capped KTO films on Si substrate. For STO/KTO/Si trilayer, the lattice mismatch is 3.6% for STO/KTO on Si substrate, and then *ε*_s_ is −3.6% and the Rashba spin splitting energy is 51 meV, with the *k* vector offset being 0.16 Å^−1^. It is clear that the Rashba effects are still very strong after capping layers and/or substrates are added.

**Fig. 6:**
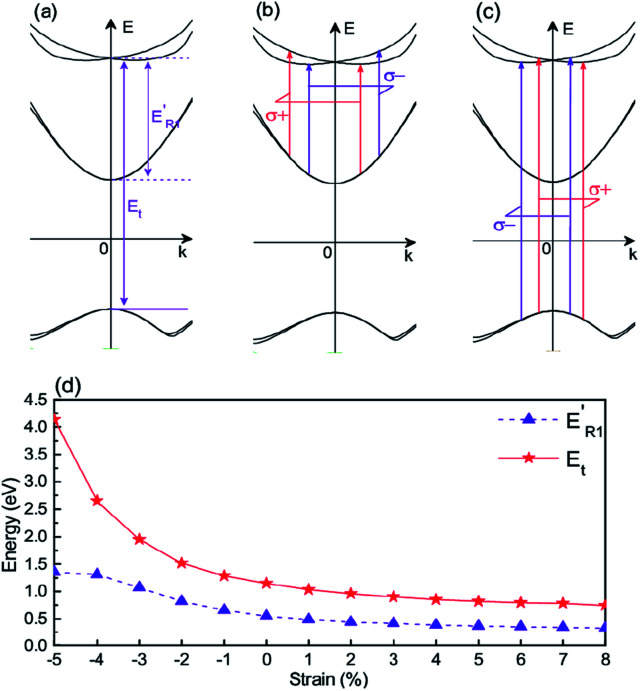
Schematic for CPGE: photon energy for transition from the valence band edge (*E*_t_) or from the conduction band edge 
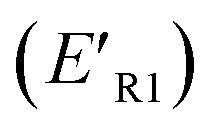
 to the Rashba bands (a); the two permitted transitions (only two *k*_*x*_ values, *k*_*x*_^±^, for a given *k*_*y*_) from the conduction band edge through circularly polarized light with *σ*_+_ (red) or *σ*_−_ (purple) (b), and the similar transitions from the valence band edge (c); and the strain dependence of the needed photon energies 
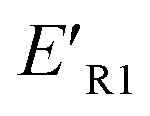
 and *E*_t_ (d).

Because of the giant Rashba spin splitting, the KTO ultrathin films can be used for achieving circular photogalvanic effect (CPGE) to generate spin-polarized photocurrents.^18–23^ For the right-handed (left-handed) circularly polarized light, its photon has the angular momentum of +1 (−1), labeled by *σ*_+_ (*σ*_−_), and the selection rule for necessary transitions is that the allowed *z*-component change of the total angular momentum is Δ*J*_m_ = +1 (−1). The valence band edge, originating from the *J* = 3/2 states, has *J*_m_ = ±3/2, and the conduction band edge, from the *d*_*xy*_ states of *l*_m_ = 2 and −2, consists of *J*_m_ = ±3/2 and ±5/2 states. Because the *d*_*yz*_/*d*_*xz*_ bands have *l*_m_ = 1 and −1, the Rashba split bands consist of *J*_m_ = ±3/2 and ±1/2 states. Therefore, for achieving the CPGE, the electrons can transit from the valence band top (with photon energy *E*_t_) or the filled conduction band edge 
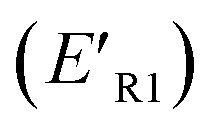
 to the *d*_*yz*_/*d*_*xz*_-based bands with giant Rashba spin splitting as the final states, as shown in Fig. 6(a). When the electron concentration is small, 
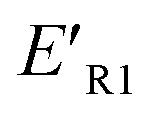
 is a little lower than *E*_R1_.

For generating a net spin-polarized photocurrent, both circularly-polarized light and the Rashba split bands are necessary.^18^ Upon illumination with a circularly polarized light with photon energy ℏ*ω* and given helicity, the energy and angular momentum conservations require that the transition happens only at the two asymmetric *k* values: *k*_*x*_^+^ and *k*_*x*_^−^.^18^ This makes the average electron velocity in the excited state become nonzero and the contributions of *k*_*x*_^±^ photoelectrons to the current do not cancel each other.^18^ Changing the photon helicity from +1 to −1 inverts the current because the “center-of-mass” for this transition is shifted in the opposite direction. This results in the generation of the spin polarized CPGE current of the Rashba split *d*_*yz*_/*d*_*xz*_ bands, as shown in Fig. 6(b and c). The photon energies needed for the CPGE from the valence band edge (*E*_t_) and the conduction band edge 
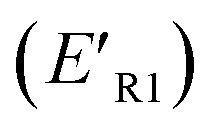
 are shown in Fig. 6(d). In principle,^18,57,58^ it can be described by *j* = *γêE*^2^*P*_circ_, where *γ* is the second-rank pseudotensor, *E* is the amplitude of the electric field of the light, *ê* is the unit vector pointing in the direction of the light propagation and *P*_circ_ is the helicity of the light beam, and the pseudotensor *γ* has non-zero element for the *C*_4v_ point group, which results in a non-zero spin-polarized photoelectron current.^19,59,60^

## IV. Conclusion

In summary, through the first-principles calculations, we have systematically investigated the effect of the biaxial stress on the Rashba spin splitting of the KTO slabs for modelling strained KTO (001) ultrathin films with the most stable surfaces.^33,34^ The calculated results reveal that tensile in-plane strain changes the Rashba spin splitting little, but the Rashba spin splitting energy *E*_R_ increases with compressive stress increasing, which is in reasonable agreement with recent experimental result. When the compressive in-plane strain approaches *ε*_s_ = −5%, *E*_R_ is 140 meV and the *k* vector offset is *k*_R_ = 0.21 Å^−1^, which corresponds to a large Rashba coupling constant 1.3 eV Å. Our analysis indicates that the enhanced giant Rashba spin splitting energy can be attributed to the strong out-of-plane electric field generated by the large compressive biaxial stress. Our further calculations show that the Rashba spin splitting energy can be enhanced to the record 190 meV by adding a STO capping layer. We also show that such giant Rashba effect can be used to generate spin-polarized photocurrents in terms of the circular photogalvanic effect.^18–23^ Therefore, these giant Rashba phenomena may open a new door to promising spintronic and optoelectronic applications.

## Conflicts of interest

There are no conflicts to declare.

## Supplementary Material
